# Farnesoid X Receptor Alleviates Cisplatin‐Induced Kidney Inflammatory Injury by Inhibiting Tlr4/NF‐κB Pathway

**DOI:** 10.1111/jcmm.70730

**Published:** 2025-07-27

**Authors:** Fangyuan Peng, Jinghan Feng, Xinni Zhang, Ting Ren, Qi Zeng, Qian Sun, Zhouping Zou, Xiaoqiang Ding, Ping Jia

**Affiliations:** ^1^ Division of Nephrology, Zhongshan Hospital Fudan University Shanghai China; ^2^ Shanghai Medical Center of Kidney Shanghai China; ^3^ Kidney and Dialysis Institute of Shanghai Shanghai China; ^4^ Kidney and Blood Purification Laboratory of Shanghai Shanghai China; ^5^ Hemodialysis Quality Control Center of Shanghai Shanghai China

**Keywords:** acute kidney injury, cisplatin, farnesoid X receptor, inflammation

## Abstract

Inflammatory responses play a critical role in cisplatin‐induced acute kidney injury (AKI). Farnesoid X receptor (FXR) has been shown to mitigate kidney dysfunction, but its mechanism remains unclear. This study aims to explore whether FXR reduces cisplatin‐induced AKI by modulating inflammation. Using a mouse model of AKI, we demonstrated that cisplatin‐induced obvious inflammation in the kidney, evidenced by increased macrophage and neutrophil infiltration, elevated expression of pro‐inflammatory cytokines, including interleukin‐1 beta (IL‐1β), IL‐6, C‐X‐C motif chemokine ligand (CXCL) 1, 2, 5, 20, and C‐C motif chemokine ligand (CCL) 2, and activation of the toll‐like receptor 4 (Tlr4)/nuclear factor‐kappa B (NF‐κB) pathway. RNA sequencing further corroborated these findings, revealing upregulation of inflammation‐related genes and activation of several inflammatory pathways in the kidney after cisplatin administration. Pretreatment with GW4064 (a FXR agonist) reduced inflammatory cytokine expression, immune cell infiltration, and Tlr4/NF‐κB activation, alleviating kidney injury. However, proximal tubule‐specific FXR knockout worsened renal inflammation and increased NF‐κB activity. In vitro, GW4064 decreased pro‐inflammatory cytokine production, suppressed Tlr4/NF‐κB signalling, and reduced apoptosis in cisplatin‐treated renal tubular epithelial cells. Together, these findings demonstrate that FXR significantly alleviates cisplatin‐induced renal inflammation via suppressing Tlr4/NF‐κB signalling. FXR activation may represent a promising therapeutic strategy to mitigate cisplatin‐induced AKI.

## 1 Introduction

Acute kidney injury (AKI) is marked by an accelerated and substantial reduction in renal function over a brief period, stemming from structural or functional kidney impairment. Cisplatin (Cis), an efficacious antineoplastic agent, is limited in its clinical application due to severe adverse effects, particularly nephrotoxicity. The incidence of AKI in cisplatin‐treated patients is approximately 20%–30%, with a mortality rate reaching up to 50% among these cases [1]. The primary pathological characteristics of cisplatin‐induced AKI encompass proximal tubular damage, inflammatory response, oxidative stress, and renal vascular injury. It is well documented that inflammation and apoptosis in renal tubular epithelial cells are major drivers in contributing to the onset of cisplatin‐induced AKI [2, 3]. Consequently, mitigating inflammation and apoptosis could serve as a key strategy in alleviating cisplatin‐induced nephrotoxicity.

During the progression of cisplatin‐induced AKI, damage to renal tubular epithelial cells causes a marked elevation in various levels of pro‐inflammatory chemokines and cytokines, namely C‐C motif chemokine ligand 2 (CCL2), C‐X‐C motif chemokine ligand 1 (CXCL1), CXCL2, and interleukin‐1 beta (IL‐1β) [4]. This increase initiates the recruitment of neutrophils and macrophages [5]. Neutrophils, as early immune responders at the site of kidney injury, contribute to the amplification of inflammation and tissue damage through the emission of diverse cytokines and chemokines involved in inflammation. In addition, neutrophils facilitate the inflammatory response and cellular damage by releasing neutrophil extracellular traps during AKI [6, 7]. In the acute phase of cisplatin‐induced AKI, macrophages contribute to tissue damage by phagocytosing damaged cells and secreting pro‐inflammatory cytokines [8, 9]. However, during the recovery phase, they promote renal repair by releasing anti‐inflammatory cytokines and facilitating tissue regeneration [10]. Studies have demonstrated that inhibiting macrophage recruitment reduces cisplatin or endotoxin‐induced organ injury [11, 12], underscoring the pro‐inflammatory and damaging role of macrophages during the acute phase of AKI.

Farnesoid X receptor (FXR), included in the ligand‐activated nuclear receptor superfamily, operates as a bile acid‐binding transcription factor [13]. Research has shown that FXR serves a key role in managing inflammatory processes and apoptosis. FXR attenuated neuroinflammation through interleukin‐10 (IL‐10)‐dependent promotion of macrophage type 2 (M2) macrophage polarisation and suppression of T cell‐mediated autoimmunity [14]. Additionally, FXR activation inhibited the expression of inflammatory factors and suppressed NF‐κB pathway‐mediated liver inflammation [15]. However, the precise impact of FXR on inflammation and apoptosis in cisplatin‐induced AKI remains incompletely elucidated.

In our previous study, we performed transcriptome analysis in FXR‐overexpressed renal tubular epithelial cells, and demonstrated that FXR overexpression markedly reduced the levels of cisplatin‐induced pro‐inflammatory signalling molecules [16], suggesting FXR's role in mitigating inflammation in the kidney. This present research aims to further investigate whether FXR can inhibit cisplatin‐induced renal inflammatory injury, and elucidate the underlying mechanisms through which FXR regulates the inflammatory response.

## 2 Materials and Methods

### 2.1 Animals and Animal Experiments

All experiments received approval from the Animal Care and Use Committee of Fudan University and were conducted in accordance with the National Institutes of Health Guidelines for the Care and Use of Laboratory Animals. Male C57BL/6J mice were sourced from the Animal Resource Center of Fudan University. Kap‐Cre (B6.Cg‐Tg (Kap‐icre) 29066/2Sig/J) and FXR^flox/flox^ (B6.129X1(FVB)‐Nr1h4tm1.2Gonz/J) mice were supplied by the Jackson Laboratory (Bar Harbor, ME, USA). The Kap‐Cre mice express improved Cre recombinase (iCre) regulated by the mouse kidney androgen‐regulated protein (Kap) promoter, with Cre recombinase expression in the renal proximal tubule cells of male mice. FXR^flox/flox^ Kap‐Cre (FXR‐KAP) mice were generated using the Cre‐loxP system by crossing FXR^flox/flox^ mice with Kap‐Cre mice. Genotype validation was performed by polymerase chain reaction (PCR) analysis. As shown in Figure S3A, the 380 bp band represents the wild‐type (WT) and the 300 bp band represents FXR^flox/flox^ alleles. In Figure S3B, the 236‐bp band represents the Kap‐cre transgene that is present in the proximal tubule‐specific KO mice. Additionally, we conducted immunohistochemistry staining for FXR, which demonstrated the absence of FXR expression in the proximal tubules of FXR‐Kap mice (Figure S3C), confirming tissue‐specific FXR deletion. Homozygous pups presented with standard body size and weight, displaying indicators of good health. For the animal experiments, adult mice (8–10 weeks of age) were utilised and maintained on a standard rodent chow diet.

To induce AKI, C57BL/6J mice were administered a dose of 30 mg/kg cisplatin, while FXR‐Kap and WT mice underwent injection with 20 mg/kg cisplatin. The control group was given the same dose of normal saline. To activate FXR in mice, the animals were intraperitoneally injected with the FXR agonist GW4064 (278779‐0309; Sigma, 30 mg/kg/day), formulated in a mixture of 10% dimethyl sulfoxide (DMSO) and 90% corn oil, for 1 week prior to cisplatin administration.

### 2.2 Cell Culture and Treatment

Mouse renal tubular epithelial cells (mTEC) were obtained from BeNa Culture Collection (BNCC243560). Cells were grown in 1640 medium with 10% fetal bovine serum (FBS) at 37°C under 5% CO_2_ and humidified conditions. mTECs were cultured in 6‐well plates at a density of 1 × 10^5^ cells/well and left to adhere for 12 h. When reaching 70% confluence, the cells were treated with cisplatin at a concentration of 20 μM for 0, 6, 12, and 18 h to investigate time‐dependent cytotoxicity. Control cells received an equivalent volume of vehicle (saline). To activate FXR, mTECs were treated with 5 μM GW4064 (HY‐10029, MCE) for 12 h, while control groups were subjected to an equivalent dose of DMSO.

### 2.3 RNA Sequencing and Data Analysis

As described previously [17], total RNA was isolated from the kidneys of mice treated with either normal saline or cisplatin (*n* = 3) using TRIzol reagent (Invitrogen, Carlsbad, CA). For each sample, 1–2 μg of total RNA was used for RNA sequencing library preparation. Enrichment of total RNA samples was performed using oligo dT (for rRNA removal), followed by library construction using the Kapa stranded RNA‐Seq library prep kit (Illumina). The quality of the constructed library was assessed using an Agilent 2100 Bioanalyser and quantified via the quantitative PCR method. These libraries were subsequently sequenced on an Illumina Novaseq 6000 sequencer. Image processing and base recognition were conducted by Solexa pipeline version 1.8 (offline base caller software, v. 1.8). Hierarchical clustering was utilised to visualise the differential mRNA expression profiles between normal saline‐treated and cisplatin‐treated mice. Pathway analysis was performed using the Kyoto Encyclopedia of Genes and Genomes (KEGG) to determine significant pathways among the differentially expressed mRNAs.

### 2.4 Histopathological Examinations and Serum Creatinine Analysis

Kidney sections underwent haematoxylin and eosin (H&E) staining and were subsequently examined through light microscopy to assess pathological changes. Histologic injury scores were evaluated under light microscopy using a scoring system. The percentage of morphologic changes was scored as follows: no injury (0), less than 25% (1), less than 50% (2), less than 75% (3), and more than 75% (4), as described previously [18]. Serum creatinine levels were quantified by the QuantiChrom Creatinine Assay Kit (BioAssay Systems, Hayward, CA), following the guidelines provided by the manufacturer.

### 2.5 Immunohistochemistry Staining

Kidney tissues, preserved in 10% formalin, were processed with paraffin embedding and sectioned at 4 μm thickness. The sections were deparaffinised in xylene and rehydrated through graded ethanol. Antigen retrieval was conducted by boiling sections in citrate buffer (pH 6.0) for 20 min. Endogenous peroxidase activity was inhibited by treatment with 3% hydrogen peroxide for 10 min. The sections were then blocked with 5% bovine serum albumin in phosphate‐buffered saline for 1 h and incubated for 12 h at 4°C with primary antibodies against F4/80 (521204, R&D Systems) or Lymphocyte Antigen 6 Complex, Locus G (Ly6G, ab238132, Abcam). Following washing, the sections were incubated with biotinylated secondary antibodies for 1 h at ambient temperature and then incubated with horseradish peroxidase (HRP)‐conjugated streptavidin for 30 min. 3,3′‐Diaminobenzidine substrate was utilised for colour development, and sections were counterstained with haematoxylin. Images were captured using an Olympus BX53 microscope.

### 2.6 Real‐Time PCR

Total RNA was isolated from renal tissues and mTEC cells by TRIzol reagent (Invitrogen). The extracted RNA was reverse transcribed into complementary DNA using the PrimeScript RT Reagent Kit (TaKaRa, Kyoto, Japan). Quantitative Real‐Time Polymerase Chain Reaction (qRT‐PCR) was performed using SYBR Premix Ex Taq II (Cat. No. DRR081A, TaKaRa) on a QuantStudio 3 Real‐Time PCR System (Applied Biosystems). Primers were synthesised by Sangon (Shanghai, China). Relative gene expression levels were normalised to β‐Actin and calculated using the 2^−ΔΔCt^ method. The primers are outlined in the table below.

**Table:** 

Gene name	Primer direction	Primer sequence
CXCL1 (M)	Forward (F)	CTGGGATTCACCTCAAGAACATC
CXCL1 (M)	Reverse (R)	CAGGGTCAAGGCAAGCCTC
CXCL2 (M)	Forward (F)	CCAACCACCAGGCTACAGG
CXCL2 (M)	Reverse (R)	GCGTCACACTCAAGCTCTG
CCL2 (M)	Forward (F)	TTAAAAACCTGGATCGGAACCAA
CCL2 (M)	Reverse (R)	GCATTAGCTTCAGATTTACGGGT
IL‐6 (M)	Forward (F)	TAGTCCTTCCTACCCCAATTTCC
IL‐6 (M)	Reverse (R)	TTGGTCCTTAGCCACTCCTTC
IL‐1β (M)	Forward (F)	GCAACTGTTCCTGAACTCAACT
IL‐1β (M)	Reverse (R)	ATCTTTTGGGGTCCGTCAACT
CCL5 (M)	Forward (F)	CCTGCTGCTTTGCCTACCTCTC
CCL5 (M)	Reverse (R)	ACACACTTGGCGGTTCCTTCGA
CXCL5 (M)	Forward (F)	CCGCTGGCATTTCTGTTGCTGT
CXCL5 (M)	Reverse (R)	CAGGGATCACCTCCAAATTAGCG
CCL20 (M)	Forward (F)	GTGGGTTTCACAAGACAGATGGC
CCL20 (M)	Reverse (R)	CCAGTTCTGCTTTGGATCAGCG

### 2.7 Western Blot Analysis

Kidney tissues and mTEC cells were homogenised in radio immunoprecipitation assay buffer (Thermo Fisher Scientific) supplemented with protease and phosphatase inhibitors. Protein concentrations were determined using the bicinchoninic acid protein assay kit (Thermo Fisher Scientific). Equal amounts of protein (30 μg) were separated by sodium dodecyl sulfate polyacrylamide gel electrophoresis and transferred onto polyvinylidene difluoride membranes (Millipore). The membranes were blocked with 5% non‐fat dry milk in Tris‐buffered saline with Tween 20 (TBS‐T) for 1 h at room temperature and incubated for 12 h at 4°C with primary antibodies against toll‐like receptor 4 (Tlr4, 14358, CST), p65 (8242, CST), phospho‐p65 (pp65, 3033, CST), myeloid differentiation primary response protein 88 (MyD88, 4283, CST), and β‐actin (4970, CST). Subsequently, the membranes were washed and incubated with secondary antibodies for 1 h at ambient temperature. Protein bands were detected by enhanced chemiluminescence substrate (Thermo Fisher Scientific) and imaged using a ChemiDoc XRS+ System (Bio‐Rad).

### 2.8 Flow Cytometry for Detection of Apoptotic Cells

The treated cells were resuspended in an appropriate volume of PBS at a cell concentration of 1 × 10^6^ cells/mL. A sample of 50,000–100,000 resuspended cells was centrifuged again at 1000 g for 5 min, and then we discarded the supernatant. Afterwards, we added 195 μL of Annexin V‐FITC binding buffer to gently resuspend the cells, followed by the addition of 5 μL of Annexin V‐FITC (Beyotime, Cat. No. C1062M) and 10 μL of propidium iodide (PI) staining solution (Beyotime, Cat. No. C1062M), with gentle mixing. The mixture was incubated at ambient temperature for 15 min away from light. Following incubation, the cells were washed again with binding buffer and resuspended in 500 μL of binding buffer. Samples were analysed by a flow cytometer (FACS Calibur, Becton Dickinson, Heidelberg, Germany), and data were analysed by FlowJo 10.0 software. Cells positive for Annexin V but negative for PI were identified as early apoptotic, whereas cells positive for both Annexin V and PI were classified as late apoptotic or necrotic.

### 2.9 PCR Product Analysis by Agarose Gel Electrophoresis

PCR products were separated by electrophoresis on a 1.5% agarose gel containing 0.5 μg/mL ethidium bromide. Electrophoresis was performed at 100 V for 30–40 min in 1× TAE buffer. DNA bands were visualised under UV illumination using a gel documentation system. The primers are outlined in the table below.

**Table:** 

Gene name	Primer direction	Primer sequence
FXR	Forward (F)	ATAGACAACCCCAGTGACCC
FXR	Reverse (R)	TCTAAAGGATAGCCGAATCT
KAP‐Cre	Forward (F)	AGATGCCAGGACATCAGGAACCTG
KAP‐Cre	Reverse (R)	ATCAGCCACACCAGACACAGAGATC

### 2.10 Statistical Analysis

Data are presented as mean ± standard error of the mean (SEM). Statistical analyses utilised GraphPad Prism 9 software. Differences between two groups were assessed with two‐tailed, unpaired *t*‐tests, whereas comparisons among multiple groups were conducted using one‐way ANOVA followed by Bernoulli's post‐test. A *p*‐value < 0.05 was regarded as statistically significant.

## 3 Results

### 3.1 FXR Activation Mitigates Cisplatin‐Induced Inflammatory Injury in Kidney

**FIGURE 1:**
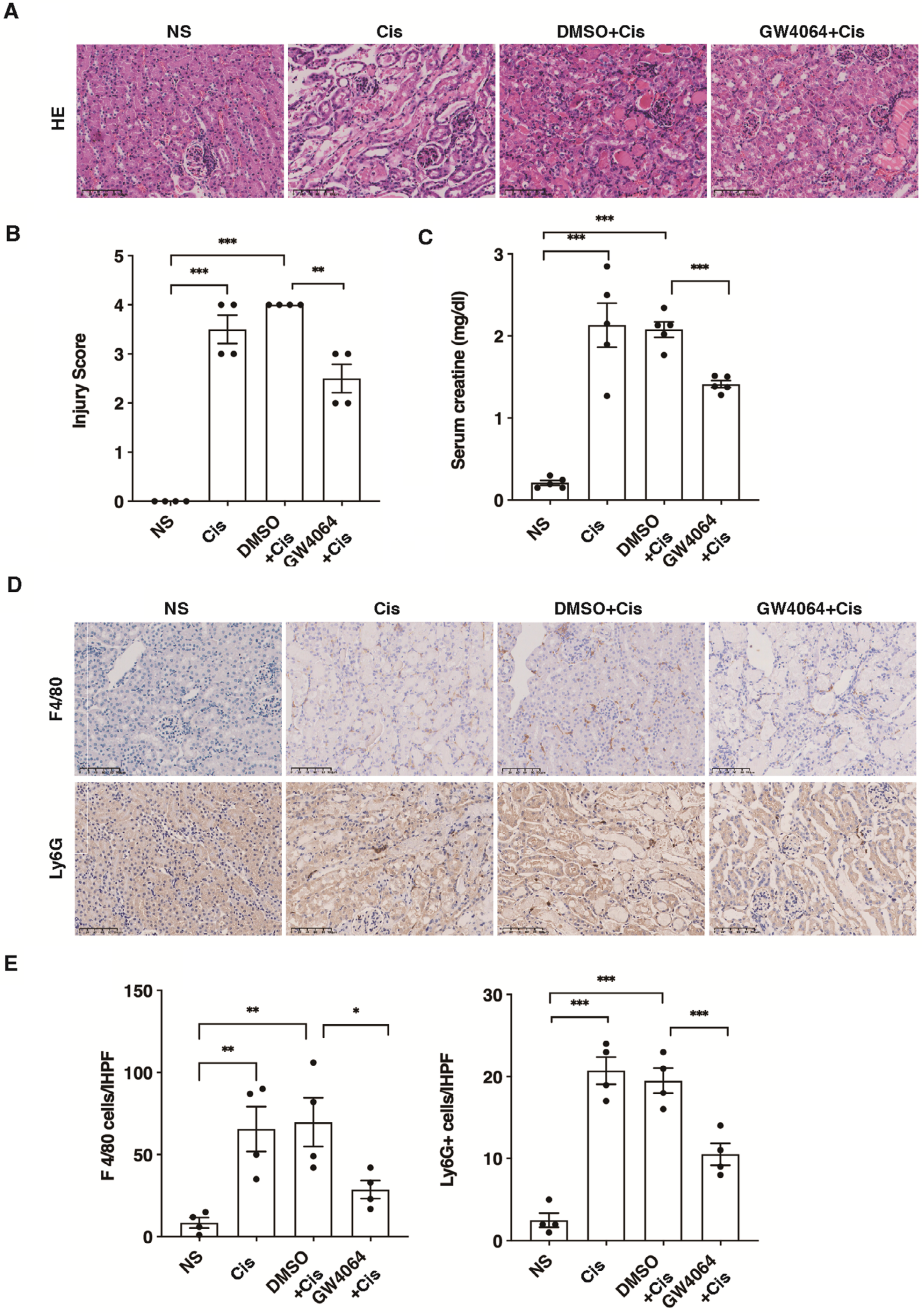
FXR activation mitigates cisplatin‐induced acute kidney injury and inflammation. Mice received either GW4064 (30 mg/kg/day) or DMSO for 5 days prior to cisplatin administration. Kidneys were collected at 72 h after cisplatin or NS administration. (A) Representative images of HE staining, F4/80 and Ly6G immunohistochemical staining in kidneys. Scale bar = 100 μm. (B) Serum creatinine levels. (C) Tubular injury scores. (D) Quantification of F4/80 positive cells. (E) Quantification of Ly6G positive cells. Data are presented as mean ± SEM. *n* = 4–5, ***p < 0.001, **p < 0.01, *p < 0.05.

We established a mouse model of AKI using a single dose of cisplatin administration (30 mg/kg). Compared to the normal saline (NS) group, the cisplatin‐treated (Cis) group showed a significant increase in serum creatinine level 72 h following cisplatin administration, and markedly renal tubular epithelial cell alterations (Figure S1A–C). These changes included cell swelling, extensive vacuolar degeneration, sporadic epithelial cell necrosis, and shedding within the tubular lumen. Immunohistochemical analysis for F4/80 and Ly6G revealed substantial macrophage and neutrophil infiltration in the Cis group compared to the NS group (Figure S1A,D,E). To assess FXR's role in the cisplatin‐induced inflammatory injury in the kidney, we used GW4064, an FXR agonist, before cisplatin injection. The results showed that FXR activation markedly mitigated renal histopathological damage in mice (Figure 1A,B). We observed an obvious reduction in serum creatinine in the mice treated with GW4064 when compared with the DMSO‐control treated mice (Figure 1C). Immunohistochemical staining showed a substantial decrease in the infiltration of macrophages and neutrophils in the kidneys of mice treated with GW4064 in comparison to the DMSO+Cis and Cis groups (Figure 1D,E).

### 3.2 FXR Activation Inhibits Tlr4/NF‐κB Pathway and Pro‐Inflammatory Cytokine Production in Kidney

**FIGURE 2:**
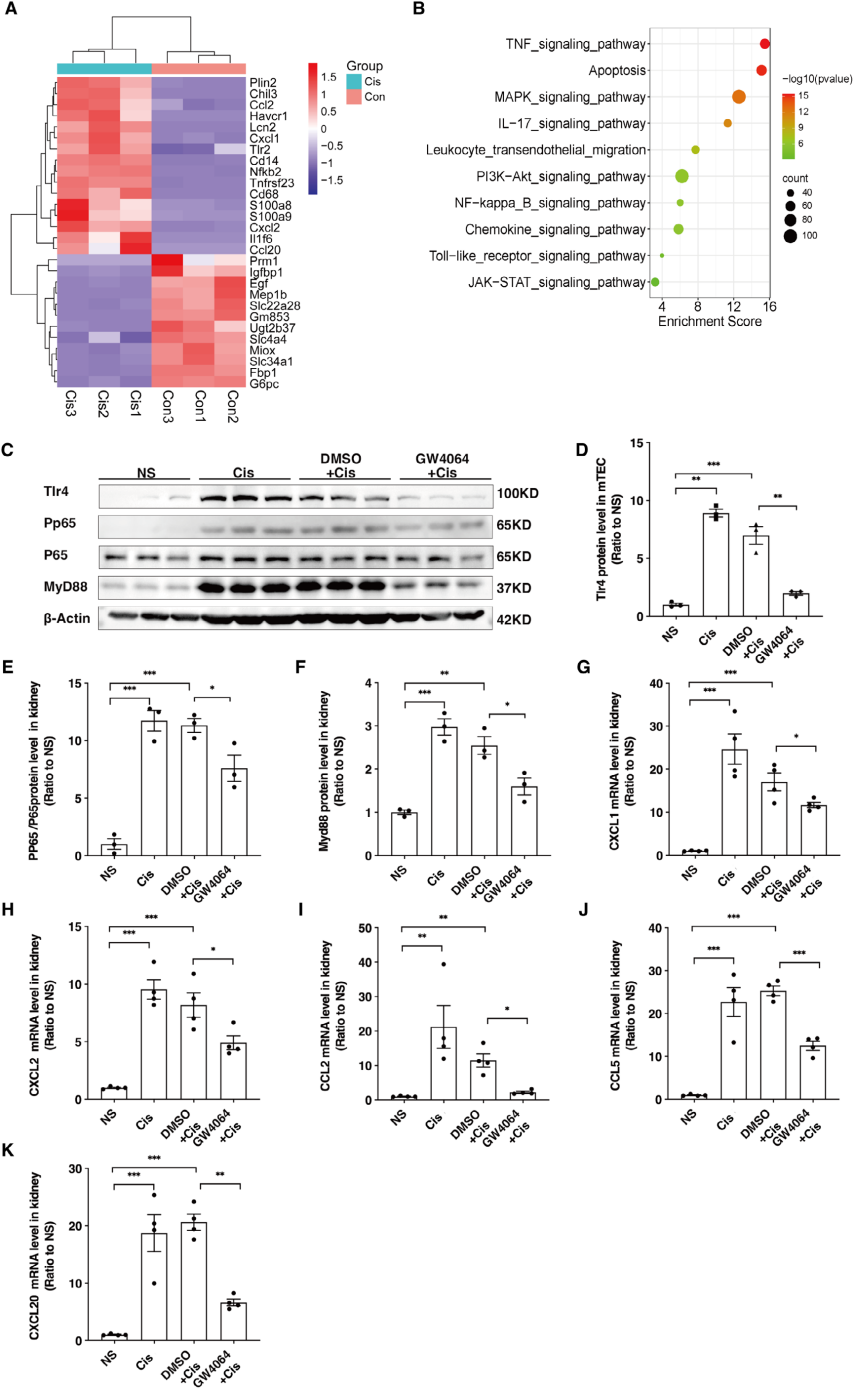
FXR activation inhibits Tlr4/NF‐κB pathway and reduces chemokine production. (A) Hierarchical clustering analysis of the differentially expressed mRNAs between Cisplatin and Control groups. Kidneys were collected at 72 h after cisplatin or normal saline administration. (B) Pathway analysis of differentially expressed genes. (C–F) Western blotting analysis of toll‐like receptor 4 (Tlr4), phospho‐p65 (PP65), and Myeloid Differentiation Primary Response Protein 88 (MyD88) expression. Data are presented as mean ± SEM. *n* = 3, ***p < 0.001, **p < 0.01, *p < 0.05. (G–K) The mRNA levels of C‐X‐C motif chemokine ligand (CXCL) 1, 2, 5, 20, and C‐C motif chemokine ligand (CCL) 2, 5, in kidneys. Data are presented as mean ± SEM. *n* = 4, ***p < 0.001, **p < 0.01, *p < 0.05.

RNA sequencing (RNA‐Seq) was performed for the evaluation of inflammation‐related gene expression and inflammatory signalling pathways in renal tissues from mice exposed to cisplatin treatment. Many inflammatory cytokines and chemokines, including CXCL1, CXCL2, CXCL20, and CCL5, were markedly elevated (fold change > 10; Figure 2A). Pathway analysis showed that genes involved in pro‐inflammatory cytokine and chemokine signalling pathways were strongly induced, including the TNF‐α signalling pathway, NF‐κB signalling pathway, and Toll‐like receptor signalling pathway (Figure 2B), indicating renal inflammation activation. Western blotting analysis showed a time‐dependent increase in Tlr4, phospho‐p65 (PP65), and Myd88 protein levels in mouse kidneys following cisplatin treatment (Figure S2A–D). RT‐PCR analysis revealed a higher level of pro‐inflammatory cytokines IL‐6, IL‐1β and TNF‐α, and higher expression of chemokines CXCL1, CXCL2, CXCL5, CXCL20, CCL2, and CCL5 in the kidneys of the Cis group compared to the NS group (Figure S2E,F), suggesting the activation of the Tlr4/NF‐κB pathway in the kidney after cisplatin administration. Subsequently, we assessed the impact of FXR on the modulation of the Tlr4/NF‐κB signalling pathway in the kidney. Western blotting analysis indicated a substantial decrease in Tlr4, MYD88, and phospho‐p65 renal expression levels of GW4064 pre‐treated mice in comparison with the DMSO+Cis and Cis groups (Figure 2A–D). The production of specific pro‐inflammatory cytokines comprising CXCL1 (Figure 2G), CXCL2 (Figure 2H), CCL2 (Figure 2I), CCL5 (Figure 2J), and CXCL20 (Figure 2K) was notably lowered accordingly in the mice pre‐treated with GW4064. These findings suggest that FXR activation inhibits Tlr4/NF‐κB signalling activation, thereby attenuating the inflammatory response during cisplatin‐induced AKI.

### 3.3 Proximal Tubule‐Specific FXR Knockout Exacerbates Cisplatin‐Induced Inflammation and Kidney Injury

**FIGURE 3:**
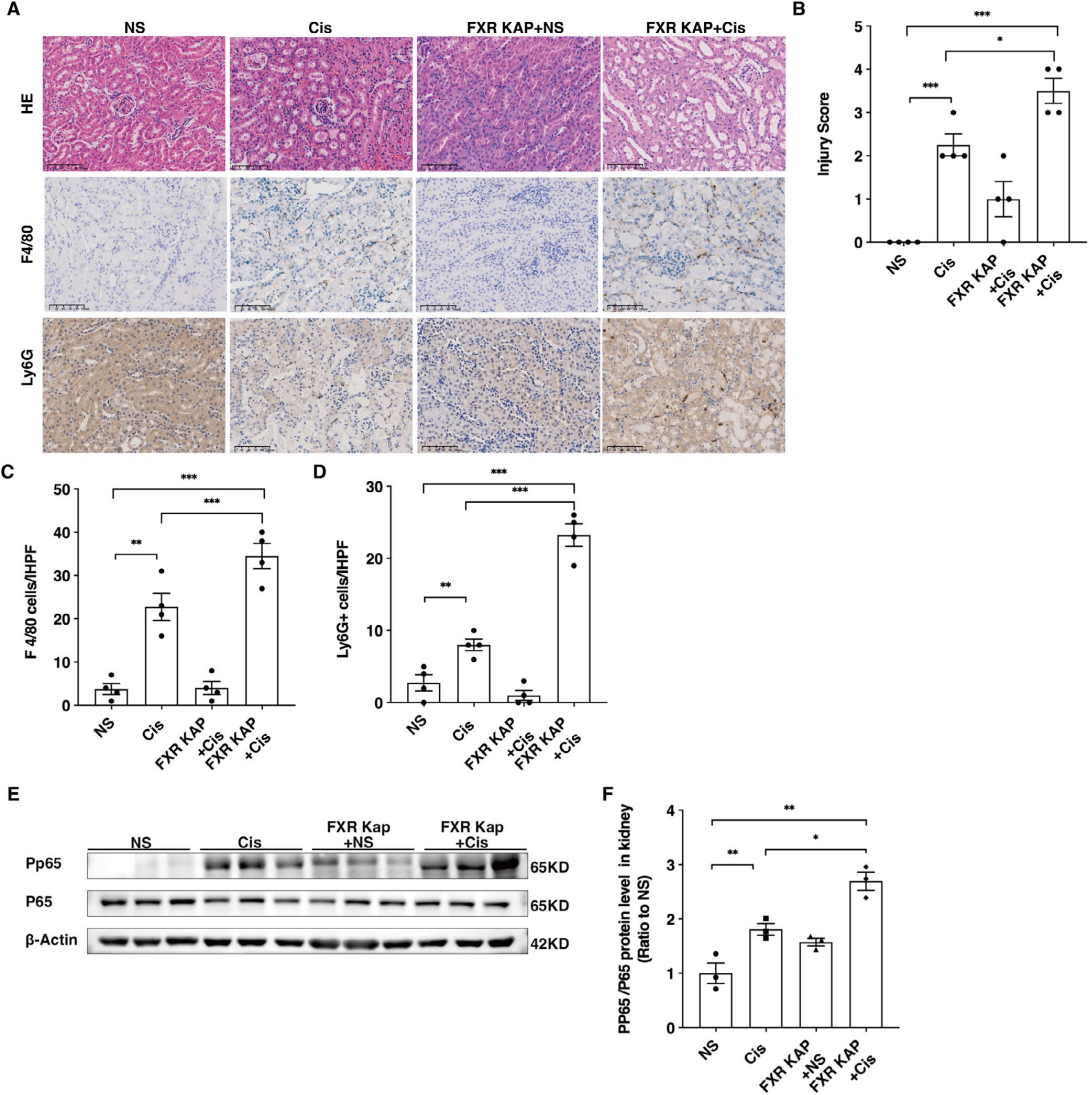
FXR knockout exacerbates cisplatin‐induced renal inflammation and acute kidney injury. Wild‐type mice and FXR‐KAP mice received either normal saline (NS) or cisplatin (Cis, 20 mg/kg) for 72 h. (A) Representative images of haematoxylin and eosin (H&E) staining, F4/80 and Ly6G immunohistochemical staining in kidneys. Scale bar = 100 μm. (B) Tubular injury scores. (C) Quantification of F4/80 positive cells. (D) Quantification of Ly6G positive cells. Data are presented as mean ± SEM. *n* = 4–5, ***p < 0.001, **p < 0.01, *p < 0.05. (E, F) Western blotting analysis of PP65 expression. Data are presented as mean ± SEM. *n* = 3, **p < 0.01, *p < 0.05.

FXR is mainly expressed by the proximal tubular epithelial cells in the kidney. To better define the function of FXR in cisplatin‐induced inflammatory injury, we generate proximal tubule‐specific FXR knockout (FXR‐Kap) mice. Male FXR‐Kap mice were utilised to establish an AKI model through the administration of 20 mg/kg of cisplatin. The results demonstrated that proximal tubule‐specific FXR knockout aggravated cisplatin‐induced renal functional deterioration and histopathological changes (Figure 3A,B). Immunohistochemistry demonstrated increased infiltration of macrophages, as indicated by F4/80 staining (Figure 3C), and neutrophils, as indicated by Ly6G staining (Figure 3D), in the kidney sections of the FXR‐Kap+Cis group relative to the WT+Cis group (20 mg/kg). Additionally, western blotting analysis confirmed substantial upregulation of PP65 protein expression in the renal tissue of the FXR‐Kap+Cis group as opposed to the WT+Cis group (Figure 3E,G). The data demonstrate that FXR absence enhances NF‐κB activity and exacerbates cisplatin‐induced renal inflammatory injury.

### 3.4 FXR Activation Reduces NF‐κB Activity and Chemokine Production in Cisplatin‐Treated Renal Tubular Epithelial Cells

**FIGURE 4:**
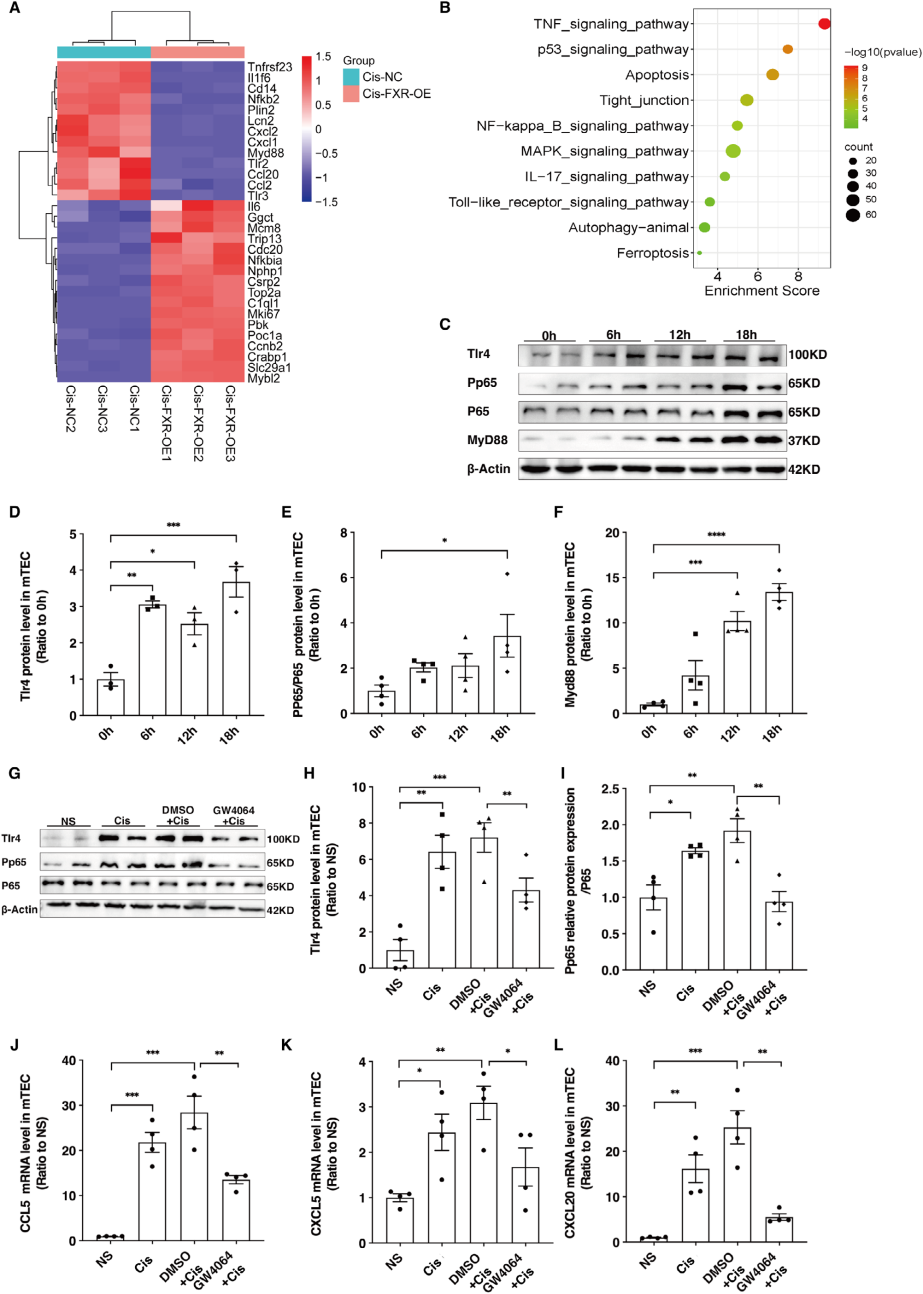
Cisplatin activates the Tlr4/NF‐κB pathway and upregulates the expression of chemokines in renal tubular epithelial cells. (A) Hierarchical clustering analysis of the differentially expressed mRNAs between Cis‐NC (negative control) and Cis‐FXR OE groups. Renal tubular epithelial cells were infected with adenovirus (Ad)‐FXRα2 (25 multiplicity of infection [MOI]) or Ad‐VP16 (25 MOI). After 36 h, the cells were challenged with cisplatin (50 m M) for 24 h. (B) KEGG pathway enrichment analysis of differentially expressed genes. (C–F) Western blotting analysis of Tlr4, PP65, and MyD88 expression at various time points after cisplatin administration in renal tubular epithelial cells. Data are presented as mean ± SEM. *n* = 3–4, ***p < 0.001, **p < 0.01, *p < 0.05. (G–I) Western blotting analysis of TLR4, PP65, and MyD88 expression. (J–L) The mRNA levels of CXCL5, 20, and CCL 5. Data are presented as mean ± SEM. *n* = 4, ***p < 0.001, **p < 0.01, *p < 0.05.

In our previous study, we conducted mRNA sequencing analysis to examine the impact of FXR overexpression in cisplatin‐treated renal tubular epithelial cells. Cells were infected with adenovirus (Ad)‐FXRα2 (25 multiplicity of infection [MOI]) or Ad‐VP16 (25 MOI). After 36 h, the cells were treated with cisplatin (50 m M) for 24 h. We observed that FXR overexpression suppressed the expression of many inflammation‐related genes, such as CXCL1, CXCL2, MYD88, and Tlr2 (Figure 4A,B). Additionally, FXR overexpression suppressed the activation of TNF‐α, apoptosis, NF‐κB, and Toll‐like receptor signalling pathways. Western blotting analysis revealed that cisplatin‐induced upregulation of the Tlr4, MYD88, and phospho‐p65 in mTEC cells (Figure 4C–F). To better clarify the function of FXR in modulating inflammation caused by cisplatin in vitro, we treated renal tubular epithelial cells with the FXR agonist GW4064 or DMSO, followed by cisplatin treatment (20 μM) for 18 h. Western blotting analysis demonstrated downregulation of Tlr4 and PP65 in the GW4064+Cis group when contrasted with the DMSO+Cis group (Figure 4G–I). In addition, RT‐PCR analysis confirmed a notable decrease in the mRNA levels of CXCL20, CXCL5, and CCL5 in the GW4064+Cis group (Figure 4J–L).

### 3.5 FXR Suppresses Apoptosis in Cisplatin‐Treated Tubular Epithelial Cells

**FIGURE 5:**
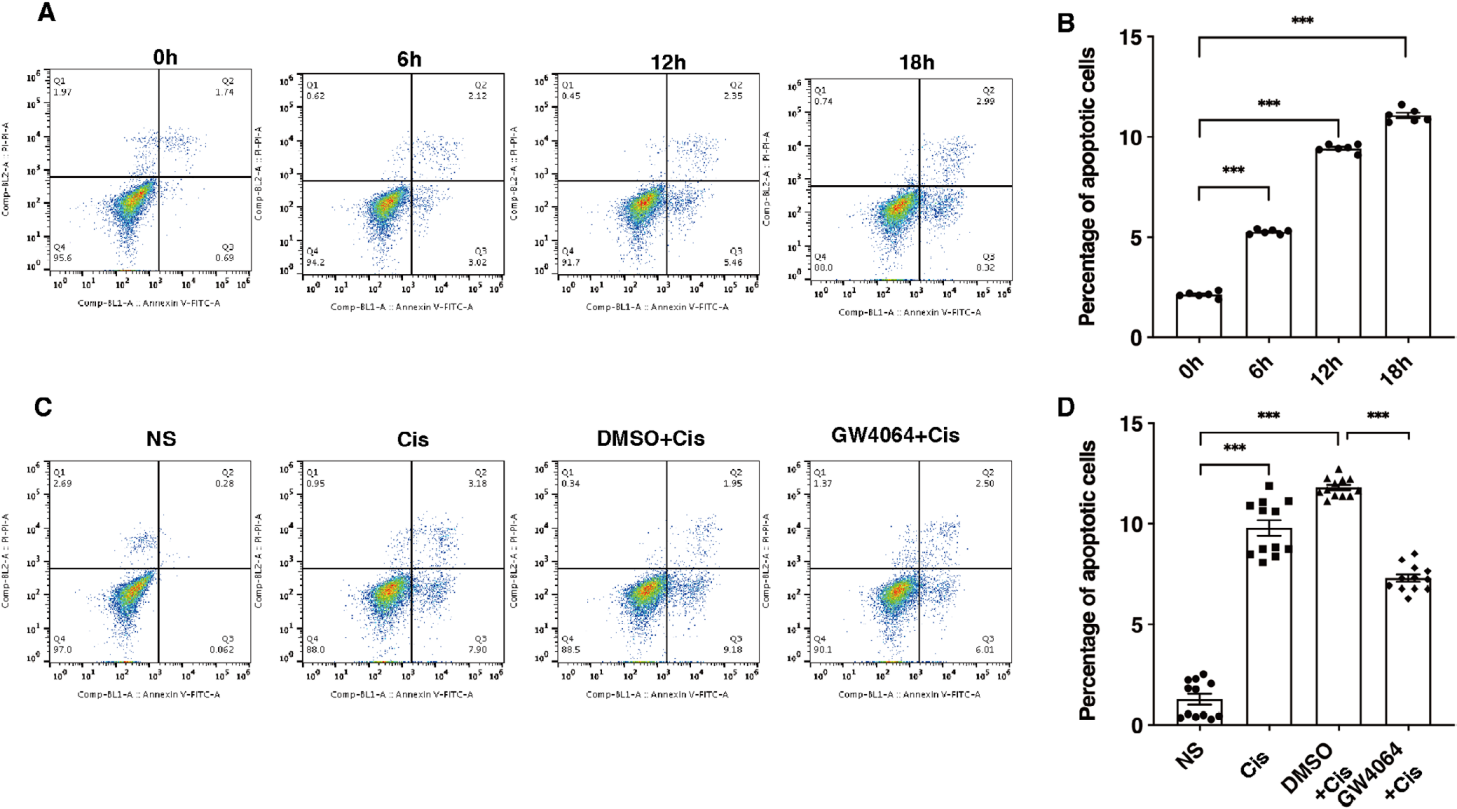
FXR activation suppresses cisplatin‐induced apoptosis in renal tubular epithelial cells. (A, B) Flow cytometry analysis of apoptosis at different time points following cisplatin treatment. (C, D) Flow cytometry analysis of apoptosis. Data are presented as mean ± SEM. *n* = 6–12, ***p < 0.001.

We exposed renal tubular epithelial cells to cisplatin (20 μM) and monitored cell apoptosis at specific designated intervals of 0, 6, 12, and 18 h. Flow cytometry analysis (Figure 5A,B) revealed a time‐dependent increase in apoptotic cell count. Then, we treated the cells with either the FXR agonist GW4064 or DMSO, followed by cisplatin treatment (20 μM) for 18 h. These findings revealed that the fraction of cells experiencing apoptosis was considerably reduced in the GW4064+Cis group in comparison with the DMSO+Cis group (Figure 5C,D).

## 4 Discussion

This study offers a detailed understanding of how FXR modulates inflammation and attenuates cisplatin‐induced renal inflammatory injury. We demonstrated that cisplatin provoked marked renal inflammation and Tlr4/NF‐κB pathway activation. Proximal tubule‐specific FXR knockout displayed more severe renal damage and inflammation following cisplatin administration. However, FXR activation markedly reduced inflammatory cytokines and chemokines' expression, suppressed the activation of Tlr4 and NF‐κB signalling axis, and attenuated cisplatin‐associated renal damage.

Inflammatory response is essential in the progression of AKI [1, 19], mediating renal damage and repair through immune cell recruitment and pro‐inflammatory cytokine and chemokine expression [2]. Among various inflammatory signalling pathways, the Tlr pathway is particularly significant in modulating immune and inflammatory response during AKI [17, 20, 21]. Tlrs, as key components of the innate immune system, detect pathogen‐associated molecular patterns (PAMPs) and damage‐associated molecular patterns (DAMPs) [22]. In AKI, DAMPs released from damaged renal cells are recognised by Tlrs, triggering downstream signalling cascades that result in pro‐inflammatory cytokine release, thereby amplifying the inflammatory response and exacerbating renal tissue injury [20]. Our previous research demonstrated that the Tlr2/NF‐κB signalling axis upregulates CCL2 in proximal tubular epithelial cells, significantly promoting inflammation and AKI during sepsis [17]. Zhang et al. revealed that Tlr4 driven nephrotoxic inflammation and tissue injury through cytokine induction, while Tlr4 inhibition reduced these effects, highlighting its potential as a therapeutic target [20]. Further research by Gao et al. demonstrated that omeprazole significantly attenuated nephrotoxicity induced by cisplatin injury via the inhibition of the Tlr4/NF‐κB/NLRP3 signalling axis [23]. Consistent with these findings, our study demonstrated the upregulation of the Tlr4/NF‐κB pathway during cisplatin‐induced AKI, with elevated levels of inflammation‐associated cytokines and chemokines, such as IL‐1β, IL‐6, CXCL1, CXCL2 and CCL2 in kidneys, suggesting the central importance of the Tlr4/NF‐κB pathway in cisplatin‐induced AKI. NF‐κB, an important transcription factor in the inflammatory response, regulates multiple genes associated with inflammation, apoptosis, and oxidative stress, making it a critical focal point for AKI research [22, 24, 25]. Fu et al. revealed that high mobility group‐1 protein promoted cisplatin‐induced kidney injury via activating NF‐κB [26]. Collectively, these findings underscore the central role of the Tlr‐mediated NF‐κB pathway in driving inflammation and tissue damage in AKI, suggesting their potential as therapeutic targets.

FXR is a member of the superfamily of ligand‐activated nuclear receptor transcription factors, primarily recognised as a bile acid‐binding transcription factor [27, 28]. FXR exhibits widespread expression across multiple organs, namely the liver, kidney, heart, and intestines, emphasising its broad physiological importance [29]. Upon activation, FXR assembles into a functional heterodimer with the retinoid X receptor α, initiating the transcription of specific target genes. This activation has demonstrated a protective role in cellular function by regulating bile acid synthesis, lipid metabolism, and glucose homeostasis [27, 30, 31, 32]. In human hepatocytes, the FXR agonist GW4064 protected against cisplatin‐induced toxicity. Furthermore, activation of FXR also protected Alexander cells against DNA‐damaging agents [33], such as mitomycin C [34]. FXR activation by GW4064 has been shown to attenuate cisplatin‐induced AKI by suppressing ferroptosis through the transcriptional regulation of ferroptosis‐related and antioxidant genes [34], In our prior study, we found that FXR activation upregulated fatty acid oxidation (FAO)‐related genes, improved FAO in proximal tubular epithelial cells, causing a reduction in lipid accumulation and mitigation of kidney damage [16]. Apart from these metabolic roles, FXR has a crucial role in modulating inflammatory responses [12, 13]. Research conducted by Hucke et al. revealed that FXR mitigated neuroinflammatory damage by promoting M2 macrophage polarisation and inhibiting T cell‐mediated autoimmunity through an IL‐10‐dependent mechanism [14]. In addition, Wang et al. showed that FXR activation reduced the levels of inflammatory cytokines and liver inflammation mediated by the NF‐κB pathway [29]. In this study, we observed that overexpression of FXR in vitro markedly inhibited the production of inflammation‐associated cytokines and chemokines induced by cisplatin. The mechanisms by which FXR activation inhibits the Tlr4/NF‐κB signalling pathway in the kidney are not yet fully elucidated. Liu et al. demonstrated that FXR activation downregulated Tlr4 expression, modulated the key components in the TLR4/NF‐κB signalling pathway, such as MyD88, phosphorylated p65, and phosphorylated IκBα, thereby contributing to the attenuation of inflammation [35]. Jing et al. found that FXR activation could inhibit NF‐κB activity, reducing the pro‐inflammatory factors in the kidney in the septic rat model [36]. Collectively, FXR displays an anti‐inflammatory property and functions as a critical modulator of inflammatory response across various tissues [37, 38].

In conclusion, our study elucidates FXR's protective function in mitigating cisplatin‐induced inflammatory response and AKI. Furthermore, our results demonstrate that FXR attenuates renal inflammation via suppressing the activation of Tlr4/NF‐κB signalling pathway. FXR activation may be a potential strategy for the prevention of cisplatin‐induced AKI.

## Author Contributions

**Fangyuan Peng:** conceptualization (equal), data curation (equal), formal analysis (equal), investigation (equal), methodology (equal), writing – original draft (lead). **Jinghan Feng:** data curation (equal), formal analysis (equal). **Xinni Zhang:** data curation (equal), investigation (equal). **Ting Ren:** data curation (equal), investigation (equal). **Qi Zeng:** formal analysis (equal), validation (equal). **Qian Sun:** data curation (equal), investigation (equal), validation (equal). **Xiaoqiang Ding:** data curation (equal), formal analysis (equal). **Ping Jia:** conceptualization (equal), data curation (equal), formal analysis (equal), funding acquisition (equal), investigation (equal), methodology (equal), project administration (equal), resources (equal), software (equal), supervision (equal), validation (equal), visualization (equal), writing – review and editing (lead). **Zhouping Zou:** writing – review and editing (equal).

## Conflicts of Interest

The authors declare no conflicts of interest.

## Supporting information


**Figure S1.** Cisplatin induces acute kidney injury and inflammation. Mice received either NS or cisplatin (30 mg/kg), 72 h later, kidneys were collected. (A) Representative images of HE staining, F4/80 and Ly6G immunohistochemical staining in kidneys. Scale bar = 100 μm. (B) Serum creatinine levels between groups. (C) Tubular injury scores. (D) Quantification of F4/80 positive cells. (E) Quantification of Ly6G positive cells. Data are presented as mean ± SEM. *n* = 4–5, ***p < 0.001, **p < 0.01, *p < 0.05.
**Figure S2.** Cisplatin activates the Tlr4/NF‐κB pathway and upregulates the expression of pro‐inflammatory cytokines in kidney. (A–D) Western blotting analysis of Tlr4, PP65, and MyD88 expression in kidneys at various time points following cisplatin injection. (E) The mRNA levels of interleukin‐1 beta (IL‐1β), IL‐6, and tumour necrosis factor‐alpha (TNF‐α) in kidneys. (F) The mRNA levels of C‐X‐C motif chemokine ligand (CXCL) 1, 2, 5, 20, and C‐C motif chemokine ligand (CCL) 2, 5, in kidneys. Data are presented as mean ± SEM. *n* = 4–5, ***p < 0.001, **p < 0.01, *p < 0.05.
**Figure S3.** Validation of proximal tubule‐specific FXR knockout. (A) Genotyping results showing that the PCR product of FXR‐floxed mice is 380 bp, while that of wild‐type (WT) mice is 300 bp. (B) Genotyping of Kap‐Cre mice showing a specific PCR product of 236 bp. (C) Immunohistochemical staining for FXR in kidney tissues, showing the absence of FXR expression in proximal tubules of FXR‐Kap mice, confirming tissue‐specific deletion.

## Data Availability

The data used to support the findings of this study are available from the corresponding author upon request.
