# Supplementary material for: Farnesoid X Receptor Alleviates Cisplatin‐Induced Kidney Inflammatory Injury by Inhibiting Tlr4/NF‐κB Pathway
Source: J Cell Mol Med. 2025 Jul 27;29(14):e70730. doi: 10.1111/jcmm.70730 (PMC12301264; doi:10.1111/jcmm.70730)
Supplement: Supplementary file 1 — Figure S1. Cisplatin induces acute kidney injury and inflammation. Mice received either NS or cisplatin (30 mg/kg), 72 h later, kidneys were collected. (A) Representative images of HE staining, F4/80 and Ly6G immunohistochemical staining in kidneys. Scale bar = 100 μm. (B) Serum creatinine levels between groups. (C) Tubular injury scores. (D) Quantification of F4/80 positive cells. (E) Quantification of Ly6G positive cells. Data are presented as mean ± SEM. n = 4–5, ***p < 0.001, **p < 0.01, *p < 0.05. Figure S2. Cisplatin activates the Tlr4/NF‐κB pathway and upregulates the expression of pro‐inflammatory cytokines in kidney. (A–D) Western blotting analysis of Tlr4, PP65, and MyD88 expression in kidneys at various time points following cisplatin injection. (E) The mRNA levels of interleukin‐1 beta (IL‐1β), IL‐6, and tumour necrosis factor‐alpha (TNF‐α) in kidneys. (F) The mRNA levels of C‐X‐C motif chemokine ligand (CXCL) 1, 2, 5, 20, and C‐C motif chemokine ligand (CCL) 2, 5, in kidneys. Data are presented as mean ± SEM. n = 4–5, ***p < 0.001, **p < 0.01, *p < 0.05. Figure S3. Validation of proximal tubule‐specific FXR knockout. (A) Genotyping results showing that the PCR product of FXR‐floxed mice is 380 bp, while that of wild‐type (WT) mice is 300 bp. (B) Genotyping of Kap‐Cre mice showing a specific PCR product of 236 bp. (C) Immunohistochemical staining for FXR in kidney tissues, showing the absence of FXR expression in proximal tubules of FXR‐Kap mice, confirming tissue‐specific deletion. [file JCMM-29-e70730-s001.docx]

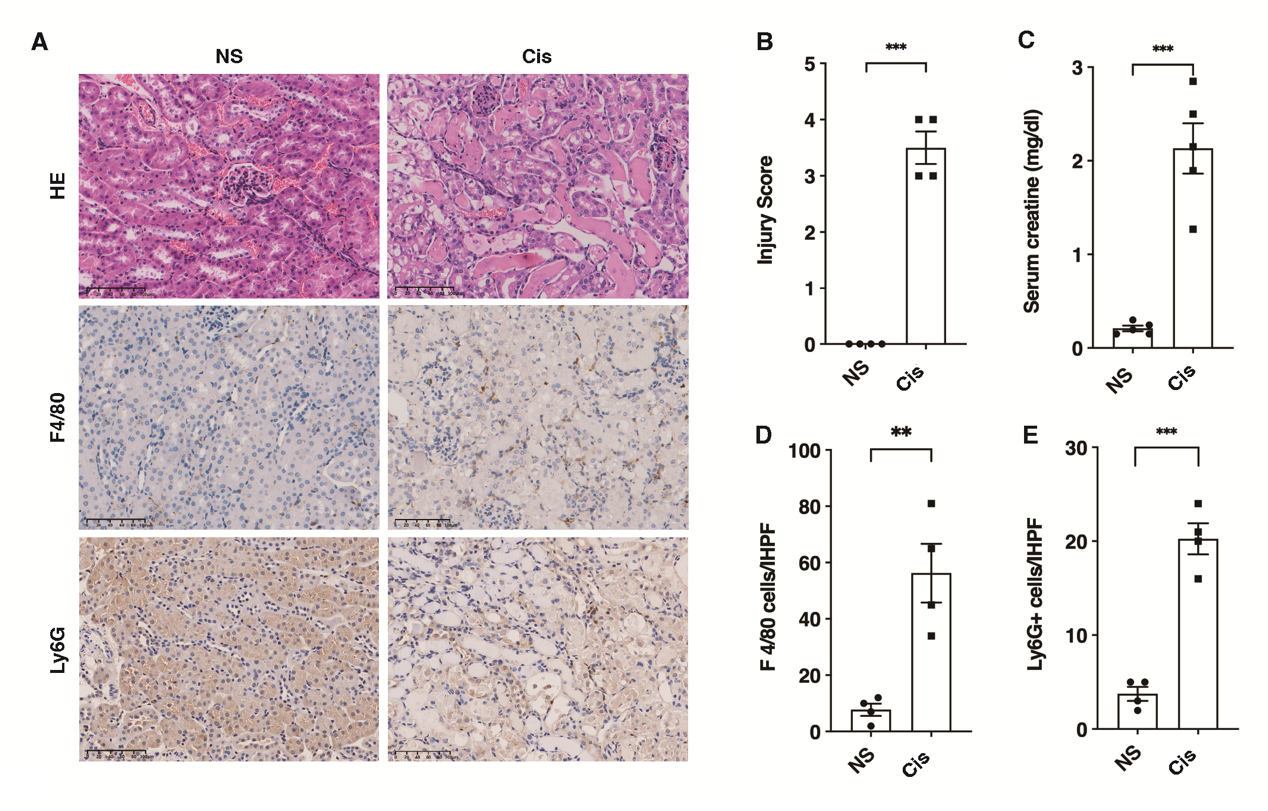


**Figure S1** Cisplatin induces acute kidney injury and inflammation. Mice received either NS or cisplatin (30 mg/kg), 72 hours later, kidneys were collected. A. Representative images of HE staining, F4/80 and Ly6G immunohistochemical staining in kidneys. Scale bar = 100 μm. B. Serum creatinine levels between groups. C. Tubular injury scores. D. Quantification of F4/80 positive cells. E. Quantification of Ly6G positive cells. Data are presented as mean ± SEM. n = 4-5, ***P < 0.001, **P < 0.01, *P < 0.05.


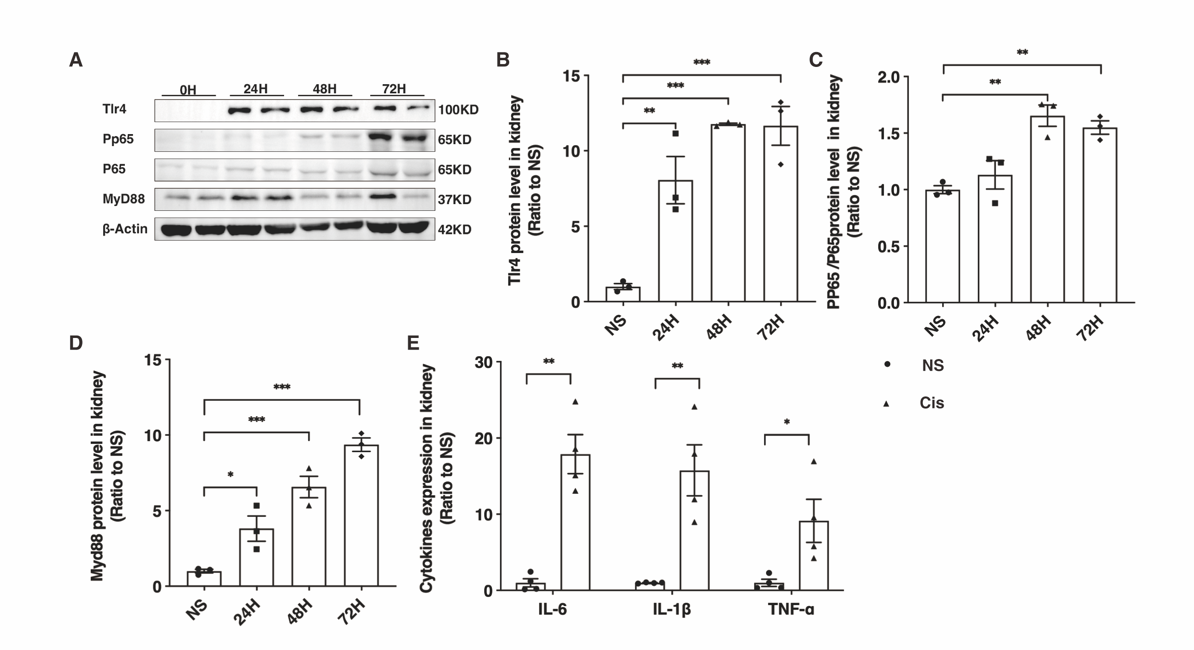


**Figure S2.** Cisplatin activates the Tlr4/NF-κB pathway and upregulates the expression of pro-inflammatory cytokines in kidney. A-D. Western blotting analysis of Tlr4, PP65, and MyD88 expression in kidneys at various time points following cisplatin injection. E. The mRNA levels of interleukin-1 beta (IL-1β), IL-6, and tumor necrosis factor-alpha (TNF-α) in kidneys. F. The mRNA levels of C-X-C motif chemokine ligand (CXCL) 1, 2, 5, 20, and C-C motif chemokine ligand (CCL) 2, 5, in kidneys. Data are presented as mean ± SEM. n = 4-5, ***P < 0.001, **P < 0.01, *P < 0.05.


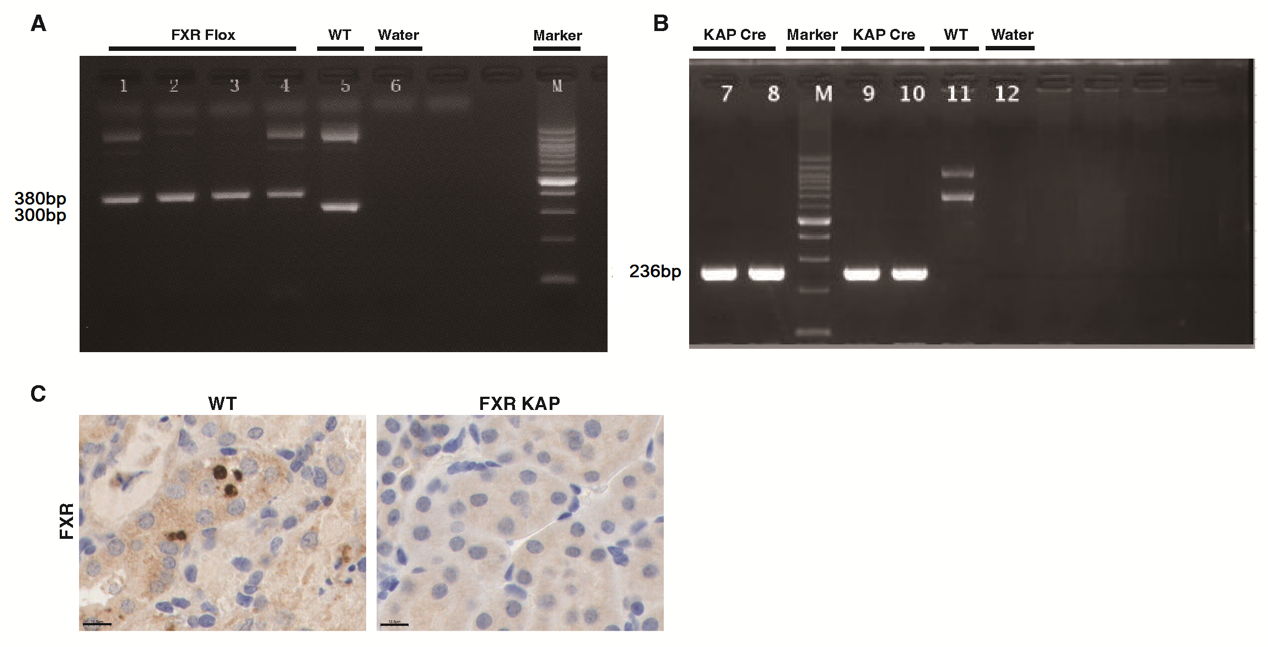


**Figure S3**. Validation of proximal tubule-specific FXR knockout. A. Genotyping results showing that the PCR product of FXR-floxed mice is 380 bp, while that of wild-type (WT) mice is 300 bp. B. Genotyping of Kap-Cre mice showing a specific PCR product of 236 bp. C. Immunohistochemical staining for FXR in kidney tissues, showing the absence of FXR expression in proximal tubules of FXR Kap mice, confirming tissue-specific deletion
